# A Review of the Impact of Microfluidics Technology on Sperm Selection Technique

**DOI:** 10.7759/cureus.27369

**Published:** 2022-07-27

**Authors:** Oluwabunmi Olatunji, Akash More

**Affiliations:** 1 Clinical Embryology, School of Allied Health Science, Datta Meghe Institute of Medical Sciences, Wardha, IND; 2 Anatomy, Jawaharlal Nehru Medical College, Datta Meghe Institute of Medical Sciences, Wardha, IND

**Keywords:** intracytoplasmic sperm injection (icsi), sperm sorting, in vitro fertilization, sperm selection, human sperm, assisted reproduction technology, microfluidic devices

## Abstract

Sperm sorting procedures depend on centrifugation processes. These processes produce oxidative stress and cell damage that are undesirable for in-vitro fertilization/intracytoplasmic sperm injection (IVF/ICSI) outcomes because they affect fertilization and implantation chances. The microfluidic sperm selection technique has shown promise in this area. It can create a platform for isolating and manipulating good-quality sperm cells using diverse triggers such as mechanical factors, chemical agents, and temperature gradients. Furthermore, microfluidic platforms can direct sperm cells for IVF or sperm sorting by utilizing an approach that is passive or active. In this review, we explain the use of microfluidics technologies for sorting and arranging sperm cells for different purposes. We also discuss the use of microfluidics technology in selecting and assessing sperm parameters and how it affects male infertility.

## Introduction and background

About 50-180 million married people worldwide are affected by infertility, and 50% of these cases are caused by female and male root causes, while about 30% are mainly caused by male factors [1]. A commonly assessed cause of infertility in males is the decline in the quality of sperm [2,3]. Andrologists usually evaluate the health of sperm based on the concentration, morphology, motility, vitality, DNA integrity, and volume, among other parameters [4]. The opportunity to get a good population of sperm for fertilization is essential for artificial insemination, in-vitro fertilization/intracytoplasmic sperm injection (IVF/ICSI), and cryopreservation of sperm [5]. Different steps of the sperm selection process for assisted reproductive techniques (ART) require several sperm washing processes [6,7]. Swim-up and centrifugation methods are routinely used during IVF procedures to remove impurities and retrieve sperm cells that are highly motile [8]. It has been suggested that using centrifugal force as a separation technique for semen can result in sperm DNA fragmentation (SDF), and increased concentrations of DNA fragmentation have been linked to high chances of miscarriage and potential recurrent pregnancy loss [9]. Hence, the availability of any other sperm preparation method that will enhance the collection of sperm cells with minimum DNA fragmentation can assist in increasing better pregnancy outcomes [10].

Microfluidic devices are delicate automated devices emerging as the platform for selecting sperm and conducting gamete function studies [11-13]. Factors such as transparency of the device, presence of an enclosed environment similar to in-vivo environment, high elaborate semen analysis, low volume size, and reduced SDF facilitate the importance of this device in ART [14,15]. Microfluidics’s attempt on the motility of sperm has displayed good feedback for swift selection of high DNA integrity sperm cells [16,17]. Studies have reported about 80% improvement in DNA integrity of sperms when microfluidics was used as the sperm selection technique against conventional methods [18]. The microfluidic device's advantage is how it mimics the in-vivo environment. As such, the media in this device for sperm selection based on motility should be similar to the cervical mucus with little or no adverse effect. Hyaluronic acid and methylcellulose are common alternatives to cervical mucus since they have been found to act similar to cervical mucus [19]. In these media, migration and survival rates and sperm motility are similar to that of the female genital tract. However, there is still a shortfall of information about the viability and motility of sperm in hyaluronic acid and methylcellulose within the highly confined compartments of microfluidic devices [20].

In-vivo fertilization requires swimming capability and persistence for spermatozoa to get to the fertilization site. In the female genital tract, the viscosity of the fluid changes, and hormonal factors and other factors trigger it. Therefore, the state of fluid viscosity influences the migration of sperm as it affects its swimming pattern and may also affect the metabolism [20]. In line with the widening use of ART worldwide, it still does not give a high success rate as there are still some compelling issues with it, such as the issues that arise from the analysis of sperm cells. Therefore, this paper aims to review the use of microfluidics in sperm selection processes and motility assessment.

## Review

Microfluidics technology

Definition

Microfluidics is a relatively young technology that dates back to the 1960s. It combines various fields of science and biotechnology to give a system that uses minimum fluid volume to integrate, automate, and manipulate high-yielding analysis. It studies fluid activities at a submicrolitre phase. This technology applies to cell biology, diagnostics, and analytic medicine [21]. Microfluidic devices handle sperm cells, oocytes, and embryos and carry out other processes in an environment similar to an in-vivo environment. A microfluidic device must integrate four main properties to be functional: the formation of droplets, sorting, expansion, and restoration [22].

Structure of Microfluidic Devices

Using the concept of laminar flow, in 2003, Cho et al. [23] built a microfluidic device for the sorting of sperm, and this was made up of four chambers: two outlets and two inlets. Due to sperm motility, motile sperm move from one point to another, thereby isolating non-motile from motile sperm cells and other cells [23]. At the same time, the stable flow of fluid was produced by a passive pump created by the differences in height between the outlet and inlet chamber [23]. With this device, they reported that the purity of motile sperm cells to the total number of sperm was higher in the outlet chamber. Since then, other studies have been carried out using microfluidic devices to sort out quality sperm cells, which are better than unsorted sperm in terms of viability, motility, and morphology [24].

With the use of multiple output channels, differences in sperm separation efficiency have been investigated, which has led to an increase in sperm viability [25]. Compared with the swim-up and centrifugation process, microfluidic devices select sperm with lower DNA fragmentation [26]. In 2017, Huang et al. proposed a six-chamber microfluidic chip with three outlet and inlet channels with a pump system that regulates the rate of laminar flow, and this led to longer stability and lower velocity in the system [25]. The cytometric flow analysis in this microfluidic device revealed that sorted sperm cells were alive and motile. Other human sperm separation chips have been developed based on the positive rheotaxis mechanism. They took advantage of this phenomenon and created a microfluidic device employing the flow generated by hydrostatic pressure [25].

Classification Based on Presentation

Based on fabrication and handling techniques, microfluidics technology can be divided into two groups: emulsion droplet and digital/droplet-based microfluidics (DMF).

1. Emulsion droplet microfluidic method: This device uses microdroplets generated from the addition of fluids such as water and oil. In its application in ART, this part of microfluidics is close to the technique used by embryologists in gamete culturing, and it is easy to handle in clinical settings [27].

2. DMF: DMF includes manipulating and creating droplets inside microdevices [28]. Because of the dominance of molecular diffusion in continuous microfluidic flow, they manipulate and control particular regions of fluid rather than the overall fluid flow. DMF has several advantages, such as shorter reaction time, improved dispersion control, separation, mixing, and reduced sample and reagent size [27]. DMF devices are compatible with many biological and chemical reagents and can perform a wide range of digital fluidic operations. In DMF, individual droplets in confined or open channels can be controlled by noncontact forces such as thermal, electrical, and magnetic forces. These non-contact pressures are extremely beneficial, particularly in ART and IVF applications. This method can potentially solve various additional biomedical difficulties for enhanced diagnostics [29,30].

Classification Based on Function

Microfluidics enables faster and easier sperm cell sorting, more accurately matching the natural selection method and avoiding some of the most dangerous parts of traditional sperm sorting procedures. Microfluidic sperm sorting techniques are roughly categorized into three types: those that isolate only motile sperm, those that separate sperm without relying on sperm movement, and those that monitor and choose individual sperm [31].

The most frequently used are type 1 microfluidic devices, which include advances that improve the swim-up approach by changing the motility screening procedure to a microfluidic device. While some of these methods can choose sufficient sperm cells for intrauterine insemination, the vast majority select a far smaller and purer subpopulation suitable for IVF [32]. In general, the subpopulation picked by these approaches is nearly 100% motile and superior in terms of DNA structure and integrity to unprocessed sperm. The type 2 microfluidic system's trapping/selection mechanism is based on sperm size, structure, or other physical characteristics rather than sperm motility [32]. These devices are not necessarily designed to capture an improved sperm subpopulation; instead, they can preserve the complete fertilization capacity of a subfertile sperm sample by randomly capturing sperm cells. Type 3 microfluidic devices take advantage of microfluidics's ability to capture and evaluate the characteristics of a single sperm cell noninvasively while retaining sperm viability [32]. 

Classification Based on Principles for Semen Fluid Analysis

Microfluidic devices include glass microchips, fluid flow, electrode gate, and electric current. They can be categorized based on their principles for semen analysis, as seen in Table 1.

**Table 1:** shows a detailed explanation of the properties and principles of microfluidic device.

Basis	Properties	Functions
Coordinated sperm swimming	Microchips are made of glass with a fluid flow and electrode gate [33].	Total density of sperm, motile sperm density
Technique of resistive pulses	Microchips made of glass with a fluid and electrical current flow through an electrode gate [34].	Velocity of sperm, volume, head and shape orientation, and frequency of tail beats
Electrical resistance	Glass microchip with microchannel composed of electrode gate, inlet, and outflow storage [35]. Microchips are made of glass with two sets of electrode ports and induced liquid mechanics [36].	Sperm density, segregation of sperm cells from other cells in the semen. Defective sperm arrangement and direction.
Colometric signal	Paper-based microchips with a chemical color scale [37].	Total sperm density, motile sperm density
Sedimentation, variable swimming pattern	Two containers with sperm buffer [38].	Total sperm density, motile sperm density

Mechanism of action

Active Selection in Microfluidic Sperm Sorting

In a normal physiological state, over 200,000,000 cells are released into the vagina during unprotected coitus. Still, only a few million sperm cells escape into the fallopian tube, and only capacitated sperm cells can eventually reach the membrane of the oocyte and attain fertilization [39,40]. This process is a long and essential journey guided by three known mechanisms: chemotaxis, thermotaxis, and rheotaxis [41,42]. In an experiment by Xie et al., they used a microfluidic system where the chemoattractant retrieved only about 10% of motile sperm cells [43]. They kept cumulus cells in a double-way channel to create a chemical gradient, and the chemotaxis of sperm was displayed by the amount of sperm cells that swam in different directions [43].

In the thermotaxis mechanism, sperm cells move up a temperature gradient, which is vital to evaluating sperm quality [44]. In an experiment by Li et al., they used a microfluidic device with an interfacial valve [11]. A temperature gradient regulated explicitly by an external thermal gradient system was generated between the spaces [45]. Here, they observed that only thermotaxis sperm moved into the higher thermal chamber while the ones, not thermotaxis moved indifferently towards both branches. A known long-range process is the rheotaxis mechanism, in which sperm move up against a flow, while thermotaxis and chemotaxis are short-range means [46-48]. Similarly, Zaferani et al. used a microfluidic tool that is not harmful and can inactively differentiate active spermatozoa from semen fluid using the rheotaxis behavior of sperm [49].

The inclusion of acoustic waves within microfluidic devices has resulted in a technology that allows for a high-yield selection of functional motile sperms. The decreased energy intensity of an acoustic field prevents/reduces potential cell membrane and DNA sabotage while maintaining accurate control on the extraction of sperm cells with intended features. Acoustic waves for sorting sperms can be modified in accordance with sperm motility and structures [50]. Therefore, these waves can be divided into bulk and surface waves [51].

Bulk acoustic waves generate compressional waves within a piezoelectric material near the microchannel that fits the frequency of the resonance of the liquid inside the microchannel, resulting in the bulk fluid with acoustic waves. Surface acoustic waves are produced by several interdigitated transducers spread across a plain surface. These waves are conveyed in and on top of the piezoelectric transducers, eliminating the necessity for excitation in the fluid resonance [52].

Passive Selection in Microfluidic Sperm Sorting

Passive selection refers to cell separation procedures based on criteria other than motility, such as density and size. When processing raw sperm, passive sperm selection based on size is crucial. This cell sorting approach is driven by cells of different sizes and densities that will encounter inertial forces of varying intensity when flowing in a circular pattern microchannel. Larger cells move to the channel's outer channel, while smaller cells remain closer to the channel's center [53]. This sorting mechanism is not cell-specific and could also be used to separate microparticles. The spiral microchannel chip is commercially available due to its adaptability, and the manufacturers produce them with various materials utilizing injection molding techniques [53].

Discussion

Before fertilization, sperm cells are processed in the laboratory during IVF. It has been shown that both swim-up and density gradient approaches improve SDF in healthy spermatozoa, with the spike in DNA damage less visible after swim-up separation [54]. Furthermore, samples incubated at 37°C and frozen sperm cells are more vulnerable to a spike in SDF over time because thawing causes an increase in oxidative stress [55].

Microfluidics has vastly increased and expanded in scope over the past three decades. The transition from continuous microfluidics to DMF has improved some of the distinctive aspects of microfluidics, such as the ability to handle a small volume of fluids, high surface-to-volume ratio, fast response, molecular diffusion, low reagent, Brownian motion, and surface forces. These distinguishing characteristics, including the ability and mobility to incorporate several elements on a single chip, make these viable means for various ART applications [56]. Nontoxic, transparent, and soft materials such as the silicon polymer polydimethyl siloxane (PDMS) are used to create microfluidic devices. It is an excellent material for living cells because of its outstanding chemical stability, as it permits gases to diffuse in and out of the medium in the chambers. The PDMS gadget is attached to PDMS-coated glass support. The channel surfaces of the device are very smooth, and sperm does not bond to the PDMS substance [57].

With the recent breakthrough of three-dimensional imaging (3D), 3D printers can now be used to manufacture microfluidic devices. Devices produced by these printers are suitable for prototyping because they can be produced much more quickly than the multistep photolithography procedure required to make a master for molding PDMS devices. Likewise, PDMS cannot be utilized to print microfluidic apparatus, and no alternative polymers are available. If 3D printing resolution, materials, and pricing eventually satisfy biomedical technology's objectives, microfluidic applications will expand fast, and the technology will also allow for the creation of devices with complex characteristics [58]. Current sperm selection technologies are typically time-consuming, detrimental to sperm integrity and quality, and produce low retrieval yields. Microfluidic systems can help improve sperm sorting technology while enhancing the results of current sorting approaches. Although no studies have shown a significantly improved IVF outcome with sperm cells picked with a microfluidic sperm sorter, but the results are very encouraging [53].

However, there are limitations to the use of this device due to its high cost and technical know-how. To overcome these hurdles, embryologists/andrologists have to work hand in hand with engineers to help fabricate prototypes that can be easy to use and readily available.

## Conclusions

Microfluidic systems have shown encouraging results for sorting sperm by using diverse on-chips with mechanical and chemical stimuli. The basis of such systems is the application of microscale fluid dynamics properties to manage the efficient movement of just motile sperms. Both stimuli and nonstimuli mechanical approaches have benefits and drawbacks. That is the reason triggers should be chosen in such a way that they do not injure the sperm cells. Therefore, the final call on which approach to adopt should depend on the application and circumstances. More research is needed to fully incorporate the concept of ARTs on microfluidic devices. Finally, the sterilizing and wrapping of this device must be improved and mechanized on a wide scale for them to be inexpensive and conveniently accessible to IVF laboratories.
